# Chronodisruption and Gut Microbiota: Triggering Glycemic Imbalance in People with Type 2 Diabetes

**DOI:** 10.3390/nu16050616

**Published:** 2024-02-23

**Authors:** María Luisa Moreno-Cortés, José Enrique Meza-Alvarado, Jaime García-Mena, Azucena Hernández-Rodríguez

**Affiliations:** 1 Laboratorio de Biomedicina, Instituto de Investigaciones Biológicas, Universidad Veracruzana, Xalapa 91190, Veracruz, Mexico; zs19015587@estudiantes.uv.mx; 2 Centro de Investigaciones Biomédicas, Universidad Veracruzana, Xalapa 91000, Veracruz, Mexico; enmeza@uv.mx; 3 Departamento de Genética y Biología Molecular, Cinvestav, Av. Instituto Politécnico Nacional 2508, CDMX 07360, Mexico; jgmena@cinvestav.mx; 4 Facultad de Bioanálisis, Universidad Veracruzana, Xalapa 91010, Veracruz, Mexico

**Keywords:** dysbiosis, Type 2 diabetes, chronotherapy, circadian rhythms, microbiota transplant, eubiosis, sleep

## Abstract

The desynchronization of physiological and behavioral mechanisms influences the gut microbiota and eating behavior in mammals, as shown in both rodents and humans, leading to the development of pathologies such as Type 2 diabetes (T2D), obesity, and metabolic syndrome. Recent studies propose resynchronization as a key input controlling metabolic cycles and contributing to reducing the risk of suffering some chronic diseases such as diabetes, obesity, or metabolic syndrome. In this analytical review, we present an overview of how desynchronization and its implications for the gut microbiome make people vulnerable to intestinal dysbiosis and consequent chronic diseases. In particular, we explore the eubiosis–dysbiosis phenomenon and, finally, propose some topics aimed at addressing chronotherapy as a key strategy in the prevention of chronic diseases.

## 1. Introduction

Humanity has always presented very particular behavioral patterns, such as eating, social, and reproductive activities, as well as patterns of rest and recreation. Most of these are aimed at survival [1]. In humans, the sleep-wake cycle is a highly studied rhythmic behavior. This biological phenomenon is determined by environmental signals, including day and night light variations and food availability. Both are basic survival processes and can be observed throughout the phylogenetic scale [2,3].

Eating and sleeping are rhythmic physiological processes. Current lifestyles, such as food restriction or, in contrast, the consumption of high-calorie and hyperlipidemic diets, as well as sleep restriction or constant artificial light exposure at night, can modify these biological rhythms, with several health consequences, such as Type 2 diabetes (T2D), obesity, metabolic syndrome or cancer, among numerous others [4].

The impacts of food restriction and/or sleep disturbances have implications at both the metabolic and digestive levels, particularly contributing to intestinal dysbiosis in humans, with a consequent higher risk of illness.

Under conditions of intestinal dysbiosis, the diversity of the microbiota tends to decrease, although not necessarily the quantity. The severity of this imbalance has been associated with numerous digestive disturbances and allergic, metabolic, and even immunological diseases. However, due to the diversity of mechanisms involved, this association does not always imply causality. Efforts have been made to determine whether dysbiosis, sleep disorders, and eating disorders are the causes or the consequences of each other.

In this analytical review, we focus specifically on the impact of sleeping and eating disorders and how they affect the microbiome, leading to intestinal dysbiosis and other conditions. We propose therapeutic strategies based on chronotherapy, aimed at the prevention of chronic diseases such as diabetes.

## 2. Circadian Rhythms

**Figure 1:**
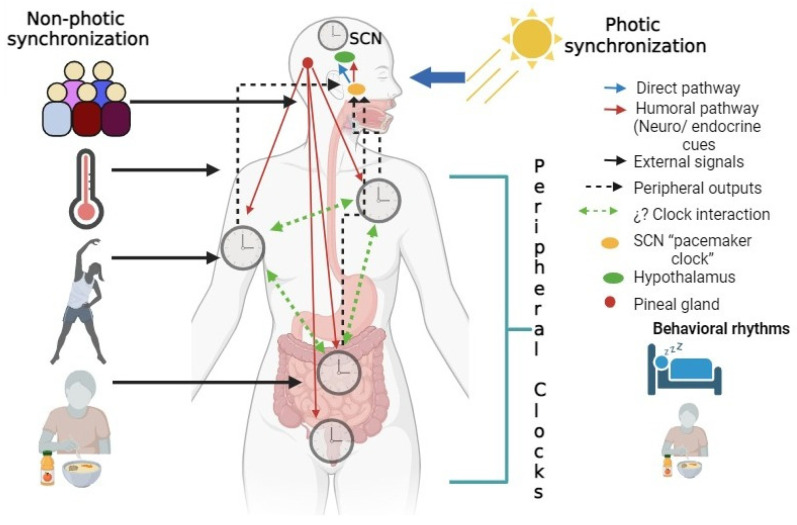
Mammalian circadian systems. Mammalian circadian clocks are organized hierarchically The master clock is the supachiasmatic nucleus (SCN) from the hypothalamus, which entrains the circadian system to the environmental light/dark cycle (photic synchronization;modulating rhythms in endocrine and behavioral processes. Photic entrainment is the main signal for synchronization. However, other external signals such as food intake, exercise, and socialization (non-photic synchronization; contribute to the synchronization of the system by generating peripheral outputs that modulate the activity of the SCN. Peripheral clocks can align with body temperature, feeding–fasting, or sleep–wake rhythms for everyday behaviors. This organization is not necessarily controlled by the SCN. The figure was created on BioRender.com.

Circadian rhythms are physiological and behavioral oscillations presented by living beings at periodic intervals of approximately 24 h. In the middle of the last century, the discovery of a biological clock in mammals (which was known as the suprachiasmatic nucleus; SCN) [5,6] opened a research theme in the biomedical area known as chronobiology. It is well known that the physiology of living beings maintains a rigorous temporal biological synchronization induced by environmental cues such as light and temperature, or behavioral stimuli such as socialization, feeding, and exercise, known as “zeitgebers” (see Figure 1). When this synchronization is broken, it induces pathophysiological alterations in the different systems within the individual, such as the metabolic, behavioral, and reproductive systems, among others.

In humans, the SCN is a group of ≈50,000 neurons [7], located bilaterally in the ventral region of the hypothalamus and dorsal to the optic chiasm. The SCN is considered the master clock in terms of the synchronization of circadian rhythms in mammals, which is entrained by the incidence of environmental light, named photic synchronization. Two efferent communication types have been studied in terms of the transmission of circadian rhythmicity throughout the entire organism: the direct way, through neural projections to other specific central structures, and a second way, known as humoral communication via molecules carried by the bloodstream and tissue fluids [8]. However, advancements in genetic research have identified that circadian rhythmicity is controlled by a molecular core, which includes a group of genes and their proteins termed clock genes. This regulating mechanism is modulated by a complex feedback loop in which the CLOCK and BMAL1 dimers bind to specific promoter regions of the *Per* and *Cry* genes, inducing their rhythmic transcription. Subsequently, PER and CRY form dimers that translocate to the nucleus, where they act to suppress the CLOCK and BMAL1 dimers, reducing their transcription [9,10]. During this process, the expression of other genes known as clock-controlled genes is induced, and thousands of genes are regulated by this mechanism [11]. Although clock genes were initially studied in the SCN, it was found that they are also expressed in an oscillatory manner in different peripheral tissues, in response to other non-photic stimuli, such as physical activity and feeding. This contributed to the proposal of a multi-oscillatory circadian system, in which the SCN is the main clock (see Figure 1).

Several studies in humans and rodents have demonstrated the participation of the circadian system in the regulation of energy balance and glucose metabolism [12,13,14]. In humans, a decrease has been found in the expression of clock genes in the pancreatic islets of diabetic patients. Moreover, this effect on the mRNA expression of *per2*, *per 3,* and *cry2* genes correlates with the insulin content of the pancreatic tissue. On the other hand, in vitro cultures of islets maintained in high glucose concentration decreased the expression of *per3* mRNA [13]. Together, these results demonstrate the relationship between the molecular circadian system and endocrine functions in regulating glucose metabolism, and how disturbance of the conditions could induce the development of pathologies, including T2D. Also, several studies have demonstrated circadian oscillations in different physiological, metabolic, endocrine, immunological, neural, and behavioral processes that participate in the homeostasis of individuals. The effects of desynchronization of these rhythms and the development of pathological alterations in different systems within the individual have also been studied. In this sense, a new concept named chronodisruption has emerged [15]. 

## 3. Chronodisruption

The term chronodisruption is defined as the disruption of circadian rhythms for long periods, inducing pathological changes in the individual. The term was coined by Erren and Reiter in 2009 [16]. This term must be differentiated from others, such as circadian disruption or chronodisturbance, in which changes are not so drastic or counteracted by periods of adaptation without generating major problems [15]. Modern lifestyles promote changes in daily habits, favoring important changes in the hours of food intake and sleep.

It has been suggested that one of the factors that favor the alterations induced by chronodisruption is that we live in modern times with ancient genomes, which are not compatible with the current photoperiods created by artificial light. It should therefore not be surprising that these drastic changes can induce metabolic perturbations [17]. 

The most representative conditions of chronodisruption induced by modern lifestyles are shift work, jet lag, social jet lag, and night eating. In all cases, characteristics include sleep deprivation or lag, changes in eating habits, an increase in body mass, and a metabolism aimed at greater energy reserves in adipose tissue. Although there are several mechanisms involved, low melatonin secretion and an increase in orexigenic hormones predominate. In parallel with chronodisruption, another new term has emerged: chronodisruptor. Erren and Reiter in 2009 [16] propose that a chronodisruptor is an exposure or effector of endogenous or exogenous origin capable of disrupting time and order. The main proposed chronodisruptor is the exposure to artificial light at night. This is due to its implications in the inhibition of melatonin secretion, which is a chronobiological hormone that modulates circadian and seasonal rhythms in mammals. Due to its broad action in different organs of the individual, melatonin inhibition is associated with the development of obesity, metabolic syndrome and cancer, as well as psychological, sleep, and reproductive disorders. 

There is a high prevalence of shift work globally, and it is estimated, in the European Union, that around 20% of the workforce works under this condition [18]. The health effects of shift work are therefore among those most studied. Several studies have revealed a clear association between shift work and metabolic disorders and a high incidence of developing general obesity, abdominal obesity, diabetes, high triglycerides, and cardiovascular abnormalities. Furthermore, tests for glucose intolerance were higher in women engaged in shift work than in day workers [19]. These effects arise because several metabolism-regulating hormones such as cortisol, insulin, glucagon, and growth hormone present circadian fluctuations, which are synchronized with their respective receptors [17]. Chronodisruption is a problem generated by modern lifestyles, that must be addressed from a multidisciplinary perspective to counteract its effects on the population.

## 4. Sleep as a Modulator of Circadian Rhythms

Several circadian rhythms are present in organisms at the endocrine level, such as the secretion of cortisol and melatonin, and physiological processes such as body temperature and the sleep–wake cycle. The sleep–wake cycle is the most evident of circadian rhythms in humans. The importance of these rhythms lies in the fact that, during periods of rest/activity, they synchronize the outputs for other endogenous rhythms of the organism [20]. Moreover, the sleep–wake rhythm is considered to exhibit the clearest sign of the oscillation of the circadian pacemaker [21]. In addition to its importance in the chronobiology of the individual, sleep is as essential to life as nutrition. When the individual is exposed to sleep deprivation, acute or chronic, or due to sleep disorders, the state of sleepiness will increase, altering the homeostasis controlled by sleep and inducing an imbalance in neurobehavioral performance [22]. It has therefore been reported that the sleep–wake cycle has two components: the homeostatic and the circadian, giving rise to the proposal of two distinct sleep processes; the “S” or homeostatic process, and the circadian “C” process. The “S” process is more neurochemical, beginning during the activity phase and is consolidated with the manifestation of sleep, which is the input of the “C” process, i.e., feeling sleepy with the “S” process will occur if it is in phase with the “C” process [23,24,25]. 

Although the pioneering studies on sleep deprivation focused on the central nervous system (CNS) functions, there is currently a large amount of information available about its negative effects on health, inducing obesity, cardiovascular diseases, cancer, and psychological disorders [26]. Although the mechanisms remain unclear, a close relationship between the homeostatic function of sleep, circadian rhythms, and metabolism is evident. Advances in molecular techniques have provided significant contributions to understanding the relationship between sleep and gene expression in different animal and human systems, with microarrays proving to be a fundamental tool. A study revealed that, in the cortex and hypothalamus of sleep-deprived mice, 2499 genes changed their expression due to sleep deprivation [27]. Similar variations were observed in the rat liver, where 426 genes were modified [28]. On the other hand, analysis of the blood transcriptome is a reference tool with which to relate gene expression with other systems in the organism [29]. A study analyzing the blood transcriptome of sleep-deprived patients revealed that 711 genes modified their expression due to the condition, and 374 changed the amplitude of their circadian expression, highlighting that some clock genes, such as those of the *per* family, and metabolic genes, such as the glucose transporter genes *slc2a3* and *slc2a14*, were altered [30]. In addition, a metabolomics study of healthy young people, in which 170 plasmatic metabolites were analyzed, revealed that 109 presented daily rhythms with increased values during the awake phase. However, when individuals were exposed to sleep deprivation, only 66 maintained their rhythmicity albeit with decreased amplitude, while the values of 27 increased significantly [31]. Taken together, these data suggest the importance of sleep as a homeostatic regulator of different physiological processes in the body.

## 5. Circadian Rhythms in the Enteral System

Several studies have shown circadian oscillations in different structural and physiological mechanisms of the gastrointestinal tract. In the 1970s, Moor and Englert reported a circadian basal secretion of gastric acids in humans [32], with low levels in the morning that increase during the day and decrease at dawn. On the other hand, intestinal motility also presents circadian patterns in humans and rodents. The gastric emptying time of solid foods in humans is faster in the afternoon, although they do not report changes in liquid contents [33]. Similarly, studies with electrogastrography have revealed that the frequency of electrical activity of the stomach increases during the afternoon [34]. In this sense, intestinal motility also oscillates daily, with increased activity presented during the day, especially after consuming food, compared with nocturnal conditions.

The innervation of the gastrointestinal tract forms part of the enteric nervous system, which is a neural network as complex as that of the spinal cord. It is divided into two main plexuses: the myenteric and the submucosal. In the rodent intestine, circadian clock gene expression has been reported in cells of the intestinal crypts and neurons of the myenteric plexus. These are synchronized by feeding time and can be uncoupled from the rhythmic expression of the central master clock located in the hypothalamus when subjects are subjected to restricted feeding [35,36,37]. Communication with the CNS is through direct sympathetic autonomic innervation in which the vagus nerve plays a major role in the communication. However, this pathway is unclear in terms of circadian coupling, given that when it was sectioned in rodents, the expression of circadian clock genes was maintained [36]. 

Microarray studies revealed that the expression of clock genes in the intestinal mucosa of patients with inflammatory bowel disease presents different patterns. In Crohn’s disease, of the 150 genes studied, 50 were expressed differently and 21 were upregulated. In ulcerative colitis, 50 genes were expressed differentially, and 27 were upregulated. It was observed that the clock genes *Arntl2* and *RorA* were upregulated, while *Csnk2B*, *Npas2*, *Per1*, and *Per3* were downregulated [38,39]. This could be related to the manifestation of these pathologies in subjects exposed to conditions of desynchronization such as shift work or jet lag [40,41].

On the other hand, early studies in rodents have shown that intestinal physiology exhibits circadian rhythms in its enzymatic activity and nutrient absorption, with greater activity presented during the awake phase in ad libitum animals, which was modified by feeding time in animals subjected to food restriction [42]. The activity of the digestive system, such as the digestion and absorption of nutrients, requires the synthesis of molecules such as enzymes and transporters. For different nutrients such as lipids [43,44], glucose [45,46], proteins [47], and electrolytes [48], regulation of their synthesis by the circadian clock has been suggested.

Early studies in rats have demonstrated that intestinal glucose absorption presents a circadian pattern regulated by the activity of its transporter, which is synchronized by feeding time [41]. The transporters SGLT1 and glucose transporter 5 (GLUT5) transport glucose to the enterocyte, and GLUT2 facilitates diffusion across the basolateral membrane. These transporters present daily fluctuations depending on the feeding habits of the species. In rodents, since they are nocturnal, their expression is elevated at night because they feed in the dark phase and persists in animals that have been deprived of food [49]. In primates, diurnal expression of the *Sglt1* gene has been reported due to their habit of eating during the light phase [45].

Interestingly, these variations are synchronized primarily by the feeding schedule, rather than the light–dark cycle [50]. Taken together, the enteric system plays an important role in glucose metabolism and its desynchronization with the circadian system, which is a fundamental factor in the development of T2D.

## 6. The Gut–Brain Axis (GBA)

In the 20th century, when several peptides were found to occur in both the brain and gastrointestinal tract, the term “gut-brain axis” was first coined, based on the prevailing concept that the brain was essential for controlling gut function [51]. 

The gut–brain axis (GBA), formed by the central (CNS) and the enteric (ENS) nervous systems, is bidirectionally correlated and coordinated by the chemical and neuronal network, responsible for maintaining tract functions, energy balance, and feeding behavior [52,53,54]. In addition, it was recently demonstrated that the microbiota, in turn, regulates circadian metabolic cycles in the brain, particularly in the SCN, hippocampus, and cortex, regions involved in learning and behavior. These structures are, therefore, also responsible for microbiota disruption [54]. One factor involved in this mechanism is blood—barrier permeability, which also exhibits circadian oscillations and is influenced by the microbiota [55]. Both the intestinal microbiota and the circadian system that resides in the CNS are innate and mature throughout the stages of life, and they can be simultaneously determined by factors such as birth route (natural or cesarean delivery), food type (breast milk or formula), and even eating behavior and sleep patterns in adulthood, which together favor or inhibit the generation of infectious, allergic, autoimmune, and chronic degenerative diseases, such as obesity, diabetes, or metabolic syndrome, which can occur at an early age or in maturity [56].

The importance of the GBA in health and diseases can be recognized by the bidirectional correlation alone: from brain to gut microbiota and vice versa. In this sense, several neuropeptides are mediators of these axes, including neuropeptide Y (NPY), calcitonin, vasoactive intestinal polypeptide, somatostatin, and corticotropin-releasing factor or calcitonin. This also involves vagal, spinal, sympathetic, and parasympathetic afferent and efferent neurons, which operate mainly via the same receptors and cellular transduction systems [51]. 

Due to their effects on health, recent attention has been paid to the central and peripheral implications of sleep and food restriction simultaneously in humans. In particular, scientific evidence supports the alteration of this bidirectional relationship in the pathophysiology of obesity and metabolic, endocrine, neural, and immune system-mediated mechanisms [57]. Obesity is typically derived from eating patterns, since the population has adopted a Western diet, and is closely tied to the microbiota [56], Moreover, in animal models with hypercaloric diets, a significant reduction of *Bacteroidetes* was observed along with an increase in *Firmicutes* [58]. Similarly, in individual humans undergoing weight-reduction diets [59], and in chronic obesity, a low inflammatory level state occurs, mediated by inflammatory cytokines and an increase in *Bifidobacterium* spp. This has also been shown to modulate inflammation in obesity [60]. The obesity phenotype is also transmissible to wild-type mice upon transplantation of the microbiota [61], although the state of health can be recovered when a sick individual receives microbiota from a healthy individual [56]. 

The GBA system plays an important role in regulating eating behaviors, balancing the energy needs of the body with hedonic impulses, and also balancing corporal weight [57]. This is due to the interaction between the hypothalamic nuclei and the orexigenic and anorexigenic system, which includes gut hormones such as ghrelin and leptin, as well as chemical signals generated by the gut microbiota or derived from the stomach or adipose tissue, respectively [62]. In addition to insulin, gut microbial metabolites, such as short-chain fatty acids (SCFAs), amino acid metabolites, and stress mediators, together regulate the metabolic needs of the individual [63], although the GBA system also displays circadian oscillations and can be entrained by intermittent fasting periods and dietary schedules for controlling food intake [57]. 

The GBA has a significant role in different aspects of physiology, including glucose homeostasis and gut motility, as well as serving as a central regulator of metabolism and appetite. To cite an example of these functions, there is an increase in the concentration of melanin and cannabinoid receptors mediated by the vagus nerve (VN) in response to a fasting condition, giving rise to an “orexigenic phenotype”, associated with an increased sense of hunger, which consequently promotes greater food consumption and weight gain. In contrast, after food intake, an “anorexic phenotype” produced by an increase in Cholecystokinin (CCK) levels and NPY receptor expression is observed. The GBA-VN action is essential for the production of insulin because it promotes hepatic glucose production. It has been reported that when vagal innervation is inhibited, hepatic glucose production is inhibited by the action of insulin, due to the central connection between the GBA and the vagus nerve. If the glucose level is higher, activity of the nerve is increased, with the opposite effect observed with higher levels of plasma insulin. Finally, it has been found that mice develop insulin resistance following vagotomy [64,65].

## 7. Microbiota and Circadian Rhythms

The bacteria, archaea, fungi, protozoa, and viruses that colonize the intestine are collectively known as the intestinal microbiota. It is estimated that at least 1000 species of bacteria can be isolated from the human intestine [66], and one gram of stool can contain 10^9^ bacterial cells [67]. The gut microbiota can encode a metagenome of trillions of genes, much larger than the millions of genes in the single human genome [68]. Currently, the effects of the microbiota on the physiology [69], immune system [70], metabolism [71], and nervous system [72] of the individual are widely known. The composition of the microbiota is dynamic and varies due to several internal and external factors, including the environment, state of development, nutritional condition, lifestyle, health condition, and the use of mainly antibiotic medication.

Given that prokaryotes were the first forms of life to arise on Earth and have been under changing environmental conditions and geophysical stimuli, such as changes in temperature and lighting, they have developed oscillatory molecular circuits to adapt to these changing conditions. Unlike mammals, studies in the blue–green alga *Synechoccus elongatus* determined that the molecular mechanism of circadian rhythms in this species is regulated by a group of genes known as *kai A*, *kai B*, and *kai C*, in which the latter is considered to be the main pacemaker of the circadian clock, allowing it to synchronize physiological mechanisms such as photosynthesis, nitrogen fixation, cell division, carbon metabolism, and gene expression [73,74]. 

On the other hand, circadian rhythms have been identified in non-photosynthetic prokaryotes, such as *Klebsiella aerogenes*, *Bacillus subtilis*, *Escherichi coli*, and members of the *Archae* family, which have been isolated from the intestinal microbiota. In this sense, the molecular mechanism of the clock has not been maintained as in the photosynthesizing cyanobacteria. However, homologous genes have emerged to regulate the clock. In the case of *B. subtilis*, it encodes Per-Arnt-Sim (PAS) domains, which is a motif sequence in the eukaryotic clock [75]. On the other hand, the *radA* gene has been characterized in *E. coli*, which is homologous to the *kaiC* gene of cyanobacteria [76]. In the case of the *Archaea* family, genes homologous to *kaiC* known as *cirA*, *cirB*, *cirC*, and *cirD* have been identified and are modulated by photic signals [77]. Recently, several studies on rodents and humans have shown that the intestinal microbiota is dynamic throughout the day, with variations in its species content and number [78,79]. Some bacteria in the human intestinal microbiota present daily rhythms of swarming and motility that are regulated by the melatonin secreted by the host in the intestinal lumen [80] and by the gut–brain axis.

Studies on mice demonstrated that hyperglycemia induced a loss of the daily rhythm in populations of bacteria of the genus *Akkermansia*, *Bifidobacterium, Allobaculum*, and *Oscillospira*, as well as a phase advance of *Prevotella*, *Proteobacteria*, and *Actinobacteria* [81]. On the other hand, studies on humans show that patients with T2D present a moderate degree of dysbiosis, and a decrease in butyrate-producing bacteria along with an increase in pathogenic bacteria, although the condition also favors some sulfate-reducing populations, which induce certain resistance to oxidative stress [82]. There is currently sufficient information to support the notion that the intestinal microbiota plays an important role in the triggering and development of diabetes, induced by changes in the bacterial population type and metabolic activity. However, it is also involved in intercommunication with the host’s metabolism, which is governed by the circadian system, regulating the expression of clock genes and changes in the physiology of the individual [83]. It is therefore suggested that the host-microbiota relationship is a symbiotic fluctuation that influences health and disease [84]. Further research is required on the clock mechanisms and the interaction between the microbiota and the host, as well as the synchronization mechanisms induced by different stimuli such as nutrient content, hormones, enzymes, or intestinal motility.

## 8. Alterations of Gut Microbiota Diversity Associated with Chronodisruption

In humans, a functional gut microbiota contributes directly or indirectly to several unseen but important physiological processes necessary for the well-being of the individual [85]. However, in many cases, it is only after proper function of the gut microbiota is adversely compromised that the physiological process in which it is involved can be recognized [86]. It has been reported that the contribution of the gut microbiota to glycemic control in humans is exerted through the modulation of bile acid transformation, short-chain fatty acid production, incretin secretion, and regulation of adipose tissue inflammation and function [87]. In an interesting study using an animal model, dietary intervention with fiber and high-grade protein improved both insulin resistance and glycemic control in streptozotocin-induced diabetic mice. The results showed that this response was mediated by an increase in the abundance of bacteria such as *Dubosiella*, *Parasutterella*, *Ruminococcaceae*, *Muribaculum*, *Allobaculum*, and *Bifidobacterium* and was largely related to amino acid metabolism, providing evidence of a functional association with the microbiota [88].

The microbial community shows a diurnal rhythmicity driven by the population dynamics of individual players and the presence of their food. *Firmicutes* are, therefore, more abundant in response to the supply of dietary glycans, and their populations decrease once the food source is exhausted. *Bacteroidetes* then dominate as they feed upon host glycans [89]. However, the microbiome rhythmicity is influenced by hormonal variations and the rhythmicity of the clock itself, as observed in female mice that show a more pronounced amplitude for *Bacteroidetes*. This gender bias was lower after *Bmal1*-deletion, detectable only at lower taxa levels, while the gut microbiota was affected in *Per1/2* mutant individuals (loss of microbial rhythms) and by Clock*∆19* (loss of microbial richness and diversity) [90]. 

**Figure 2:**
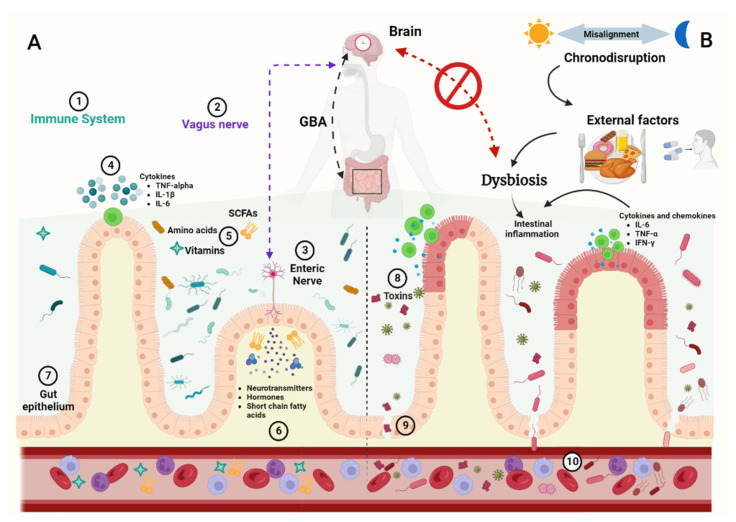
Alterations in gut microbiota diversity associated with chronodisruption. Normally, in the condition of eubiosis (**A**), intestinal microbiota presents circadian oscillations mediated by the gut–brain axis (GBA), which, in turn, mediates bidirectional relationships between the immune system (1), vagus nerve (2), and enteric communication (3), as well as microbiota metabolites such as pro and anti-inflammatory cytokines (4), amino acids, vitamins, and even short chain fatty acids (SCFAs; (5)), as well as neurotransmitters and hormones (6) that prevail in the intestinal mucosa, maintaining the integrity of the intestinal epithelial barrier (7). Otherwise, in conditions of chronodisruption (**B**), the bidirectional relationship is altered by external factors such as bad feeding habits or drug consumption, as well as toxins and pathogenic microbes (8), modifying intestinal permeability (9), passing to the bloodstream, and exacerbating dysbiosis and the inflammatory state (red epithelium; (10)). The figure was created on BioRender.com.

Chronodisruption is also associated with alterations to glycemic control (see Figure 2). In experiments conducted with mouse models under a high-fat diet, circadian rhythm disruption induced by alteration of the light/dark schedule produced mice with an average increase in fasting glucose levels of 36.2% (diabetic state) compared to the control group [91]. In humans, an extensive review of randomized controlled trials reported that sleep deprivation and poor sleep quality are linked to alterations in blood glucose as well as insulin resistance [92].

Metagenomics and relative abundance studies have revealed that the intestinal microbiota presents circadian oscillations in 60% of its population, and 10% of the commensal microbiota present diurnal oscillations [58], synchronized by feeding time and the host’s clock. Several factors induce alterations in the rhythmicity of the microbiota, including fasting, food restriction, and dietary nutrients. For example, a study on rodents observed that a high-fat diet decreases bacterial oscillation and promotes the growth of the *Firmicutes* phyla. This condition was found to improve once the animals were subjected to food restriction [57].

On the other hand, alterations in the microbiota negatively impact the quality and quantity of sleep. Dysbiosis has been reported to induce sleep fragmentation and short-duration dreams [93]. These changes have been attributed to the effect on the Hypothalamus-pituitary-adrenal (HPA) axis. Sleep fragmentation increases food consumption and the proliferation of bacteria from the *Lachnospiraceae* and *Ruminococcaceae* families, reducing the presence of the *Lactobacillaceae* family [94]. The strong relationship between the microbiota and the circadian system in mammals is notable, and any imbalance between the two will have a considerable impact on health due to dysbiosis.

Moreover, taxonomic analysis in mouse models has shown that abundances of *Firmicutes* and Proteobacteria increase during night light exposure compared to a standard light/dark (LD) photoperiod, while the ratio of *Firmicutes*: *Bacteroidetes* was higher and associated with weight gain [95]. All the evidence thus shows that chronodisruption influences the diversity and abundance of the gut microbiota.

## 9. Gut Microbiota Dysbiosis Arises from Sleep Deprivation

A review of recent reports in the field provides evidence of the association of adverse changes in gut microbiota diversity (dysbiosis) due to sleep deprivation [96]. Experiments using the murine model show that the alpha and beta diversity of the gut microbiota exhibited a significant decrease following sleep deprivation, with a reduction in SCFA production and an increase in gut inflammation with hyperpermeability [97]. The results of another study conducted with mice showed that the absence of gut microbiota suppressed the inflammatory response and cognitive impairment caused by sleep deprivation, evidencing the involvement of the gut microbiota in these physiological processes [98].

Gut microbiota dysbiosis has serious consequences for the host’s health, including obesity, T2D, and cardiovascular diseases, which represent major health problems in several countries around the world [99]. There are other diseases associated with microbiota dysbiosis, such as chronic obstructive pulmonary disease [100], Huntington’s disease [101], immune-mediated necrotizing myopathy [102], neuroinflammation and neurodegeneration [103], Alzheimer’s disease [104], Parkinson´s disease [105], and even increased susceptibility to COVID-19, as recently observed [106]. In these and many other diseases, the classic etiology may not involve a dysfunctional gut microbiota as part of the problem, but the diseases are characterized by specific microbiota signatures or alterations in the abundance of specific microorganisms. The importance of these reports is that they show that nutritional and/or chronodisruption alleviation appears to represent a therapeutic resource for the management of these diseases.

## 10. Chronotherapy, Therapeutic Effects, and Beneficial Effects by Microbiota

**Figure 3:**
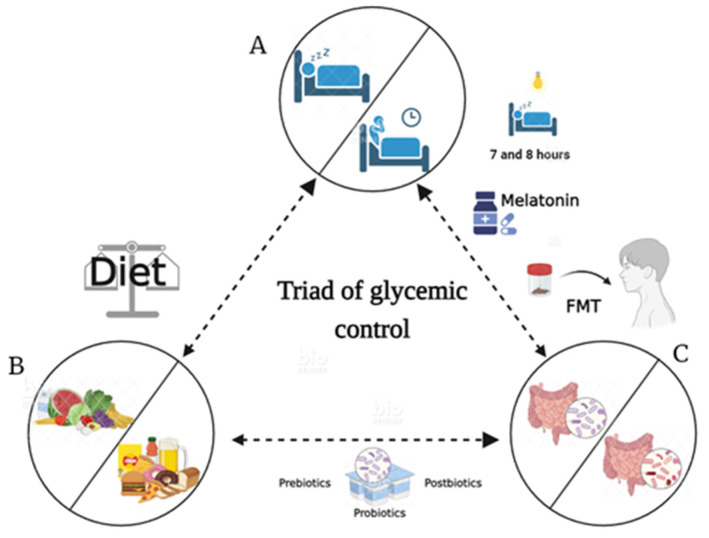
Triad of glycemic control. When chronodisruption occurs (**A**), eating habits change (**B**), dysbiosis takes place (**C**), and glycemic imbalance is accentuated in people with Type 2 diabetes. The figure was created on BioRender.com.

The diversity of gut microbiota regulates metabolism and vice versa; however, sleeping and eating patterns also play this regulatory role. In this sense, to establish a therapeutic strategy to prevent or control chronic diseases such as obesity, T2D, or metabolic syndrome, it is necessary to have therapeutic resources for the adequate management of these diseases. We propose the triad of glycemic control (see Figure 3), which aims to prevent an imbalance between sleep hygiene/sleep restriction, healthy/unhealthy dietary habits, and intestinal eubiosis/dysbiosis to avoid glycemic imbalance. 

In this context, chronotherapy seems to be an effective strategy. The goal of chronotherapy is to optimize medical treatments while taking the body’s circadian rhythms into account [107]. It is first necessary to characterize the circadian rhythms of individuals based on their chronotype, recognizing the different “chronotypes” in which physiological and behavioral rhythms range, from early (morning chronotypes) to late (evening chronotypes) [108]. 

Some reports indicate that evening chronotypes increase the probability of mortality, in which the attributed factors could be chronic misalignment between the endogenous chronobiology of the individual and externally imposed timing of work and social activities [109]. Moreover, late-chronotype individuals often skip breakfast, a practice that is associated with higher body mass indices (BMIs) and higher glycated hemoglobin A1C (HbA1C) levels [110]. Finally, it is important to consider the existence of an association between chronotype and metabolic syndrome, which is age-dependent. While early chronotypes tend to be associated with lower odds for metabolic syndrome in older individuals, they can be associated with metabolic syndrome in younger individuals [111]. 

On the other hand, to conserve glycemic control, it is necessary to improve the rhythm of sleep/wakefulness through adequate sleep hygiene and the use of chronobiotic medications such as melatonin and timed light exposure. Melatonin controls the output phase of circadian rhythms, thus modulating the circadian system. Several reports suggest that sleeping 7 to 8 h a day is a factor that reduces the risk of developing T2D. In contrast, shorter or longer durations of sleep are associated with a significantly increased risk of T2D. It should be noted that insulin resistance in young people is associated with the suppression of slow-wave sleep and rapid eye movement sleep [112]. In this regard, the importance of appropriate sleep duration to delaying or preventing T2D is highlighted [113]. The hormone melatonin is mainly recognized for its role in sleep–wake cycle regulation; however, it has also been shown to have important effects as an antioxidant and anti-inflammatory agent in diabetes. This has been demonstrated in diabetic rats treated with this hormone, presenting lower levels of inflammatory markers and a marked reduction in hepatic and extrahepatic enzyme activity [114]. 

It is also necessary to characterize the full microbiota in diverse populations, according to geographical location and socioeconomic status, to establish the “normal” human microbiota, determining whether there is a “morning” versus “night” microbiota and integrating this information into the development of chronotherapy strategies [115]. Diurnal fluctuations and intra-individual variations of the gut microbiota could be important for accurately defining states of health and implementing therapeutic strategies such as administering prebiotics, probiotics, postbiotics, and fecal transplants as part of a controlled schedule [116]. In this respect, it is necessary to first determine, according to the feeding period in which an individual is found, that the intestinal ecology and the intestinal epithelium dynamics show variations in rhythmicity, i.e., there is greater bacterial density and consequently less distance between bacterial and epithelial cells during the active feeding phase, while the opposite occurs under fasting conditions [117,118]. One example of this rhythmicity is the diurnal pattern shown by *Firmicutes* and *Bacteroidetes:* while the former is more abundant at the end of the active feeding phase, the latter increases under fasting conditions and in response to the metabolic state [78]. In summary, the gut microbiota is dynamic and rhythmic, which are important characteristics to consider when contemplating its incorporation into a therapeutic strategy [119].

On the other hand, as a risk factor for T2D, it is important to consider the diet type as part of a crucial strategy for prevention or control, considering that different eating habits and beliefs exist, such as religious fasting. Althoughthese may seem beneficial in terms of body weight, this is temporary and has a considerably mixed metabolic impact [120]. 

Fecal Microbiota Transplantation (FMT) is a novel therapeutic alternative used to manipulate the composition of the host microbiota and restore the function and structure of the gut microbiota by transferring a specific microbial community obtained from a healthy individual through specialized techniques such as colonoscopy or duodenal endoscopy [121]. This method is used for the treatment of diseases such as obesity, T2D, metabolic syndrome, and even cancer. To cite an example, FMT increases insulin sensitivity in humans and mouse diabetes models [122]. Moreover, if gut microbiota from donors with a normal BMI is transferred to obese and diabetic individuals, they present improved fecal microbiota diversity and an increase in butyrate-producing bacteria [122]. FMT treatment accompanied by dietary modifications has been found to exert great control over blood glucose and blood pressure levels, even showing potential to reverse dyslipidemia and dysarteriotony [123].

Vrieze et al. (2012) observed a notable improvement in peripheral and hepatic insulin sensitivity in individuals with metabolic syndrome after receiving an infusion of microbiota from lean donors, concluding that intestinal microbiota could be developed as therapeutic agents to increase insulin sensitivity in humans [123]. Together, these data allow us to conclude that beneficial compounds may become available to patients via FMT.

## 11. Conclusions

We show that the triad of glycemic control may prove to be an effective strategy to eliminate the imbalance betweendysbiosis, healthy and unhealthy sleep hygiene, and food restriction.

Circadian host–microbiome interactions have expanded to encompass numerous aspects of host physiology, including the fact that circadian biology is influenced not only by intestinal microbial colonization but also by sleeping and eating habits. Common pathophysiological mechanisms linked to chronic diseases such as obesity, T2D, and metabolic syndrome can be potentiated by desynchronization. Thus, resynchronization by chronotherapy can have therapeutic effects on microbiota, sleep, and eating habits, with potential benefits in terms of reducing body weight, fasting blood glucose, and HbA1C, which has potential in terms of glycemic control therapies. From this perspective, it will be fascinating to determine the temporal dynamics of microbiota, considering that the gut microbiota exhibits daily cyclical variation under a variety of dietary and feeding pattern conditions, followed by the promotion of intestinal eubiosis. For this purpose, the restoration of cyclical fluctuation of gut microflora by FMT has numerous important implications, including creating a homeostatic state, in which the host is periodically exposed to different bacterial numbers, species, and products. This could result in the coupling of corresponding rhythmicity, i.e., the FMT can act as an endogenous circadian modulator.

On the other hand, considering the observational evidence, we tend to suggest that short sleep duration and poor sleep quality are associated with insulin resistance. Moreover, both short and long sleep duration are associated with a significantly elevated risk of T2D, through changes brought about in the activity of the neuroendocrine system. In this regard, it is necessary to promote good-quality sleep to lower T2D risk. Finally, skipping breakfast was associated with an HbA1C increase from its original value. 

In order to inhibit the desynchronization effect on human health, it is necessary to avoid intestinal dysbiosis. For this purpose, and with an integral perspective, it is necessary to adjust social schedules (i.e., school and work schedules or family practices). On the other hand, promoting regular feeding schedules and maintaining a balanced dietary composition, and if necessary, applying supplementation and even interventions in behavior, such as eating patterns and sleep timing, is crucial. Finally, we proposed the triad of glycemic control to achieve a protective effect against chronic diseases, such as Type 2 diabetes.

## Data Availability

Not applicable.
